# The Association of PNPLA3 Variants with Liver Enzymes in Childhood Obesity Is Driven by the Interaction with Abdominal Fat

**DOI:** 10.1371/journal.pone.0027933

**Published:** 2011-11-28

**Authors:** Emanuele Miraglia del Giudice, Anna Grandone, Grazia Cirillo, Nicola Santoro, Alessandra Amato, Carmine Brienza, Piera Savarese, Pierluigi Marzuillo, Laura Perrone

**Affiliations:** 1 Department of Pediatrics, Seconda Università degli Studi di Napoli, Napoli, Italy; 2 Department of Pediatrics, Yale University School of Medicine, New Haven, Conneticut, United States of America; University of Granada, Spain

## Abstract

**Background and Aims:**

A polymorphism in adiponutrin/patatin-like phospholipase-3 gene (PNPLA3), rs738409 C->G, encoding for the I148M variant, is the strongest genetic determinant of liver fat and ALT levels in adulthood and childhood obesity. Aims of this study were i) to analyse in a large group of obese children the role of the interaction of not-genetic factors such as BMI, waist circumference (W/Hr) and insulin resistance (HOMA-IR) in exposing the association between the I148M polymorphism and ALT levels and ii) to stratify the individual risk of these children to have liver injury on the basis of this gene-environment interaction.

**Methods:**

1048 Italian obese children were investigated. Anthropometric, clinical and metabolic data were collected and the PNPLA3 I148M variant genotyped.

**Results:**

Children carrying the 148M allele showed higher ALT and AST levels (p = 0.000006 and p = 0.0002, respectively). Relationships between BMI-SDS, HOMA-IR and W/Hr with ALT were analysed in function of the different PNPLA3 genotypes. Children 148M homozygous showed a stronger correlation between ALT and W/Hr than those carrying the other genotypes (p: 0.0045) and, therefore, 148M homozygotes with high extent of abdominal fat (W/Hr above 0.62) had the highest OR (4.9, 95% C. I. 3.2–7.8, p = 0.00001) to develop pathologic ALT.

**Conclusions:**

We have i) showed for the first time that the magnitude of the association of PNPLA3 with liver enzymes is driven by the size of abdominal fat and ii) stratified the individual risk to develop liver damage on the basis of the interaction between the PNPLA3 genotype and abdominal fat.

## Introduction

Children and adolescents are becoming increasingly vulnerable to obesity [1]. Paralleling childhood obesity, non alcoholic fatty liver disease (NAFLD) is rapidly becoming one of the most important chronic liver diseases among children [2]. The spectrum of NAFLD ranges from simple steatosis to steatosis associated with inflammation and fibrosis (non-alcoholic steatohepatitis, NASH), which can eventually progress to liver cirrhosis [3]. It has been demonstrated, in fact, that children with NAFLD may develop later in life severe liver disease with a consequent need for liver transplantation [4]. A body of evidences shows that NAFLD is highly related to the metabolic consequences of obesity, such as dyslipidemia and insulin resistance [5], [6]. Also body fat distribution, and particularly abdominal fat, appears to be implicated in the risk to develop NAFLD [7]. A genetic predisposition, however, regardless of these risk factors, has been postulated on the basis of association and family studies [8]. Two genomewide association studies identified a polymorphism in adiponutrin/patatin-like phospholipase-3 gene (PNPLA3), rs738409 C->G, encoding for the I148M (isoleucine-to-methionine substitution at residue 148) protein variant, as the strongest genetic determinant of liver fat and alanine aminotransferase (ALT) levels [9], [10]. Adiponutrin is expressed in the liver and adipose tissue, has both triacylglycerol hydrolase and acylglycerol transacetylase activity [11] and it was suggested that the 148M allele encodes for a loss-of-function variant that predisposes to steatosis by decreasing triglyceride hydrolysis in hepatocytes [12]. Furthermore, in two series of biopsied patients the presence of the 148M allele influences both the presence of NASH and the severity of fibrosis in patients with NAFLD, irrespectively of the degree of obesity, the presence of diabetes, and the previously demonstrated effect of adiponutrin genotype on steatosis [13], [14]. The role of PNPLA3 I148M polymorphism has been recently studied also in pediatric NAFLD with results which are not univocal. Whereas Valenti el al showed an association between this polymorphism and NAFLD histologic severity [15], another study [16] found an association only with the age at the presentation of NAFLD, not supporting the clinical utility of PNPLA3 genotyping for risk stratification [17].

Interestingly, whereas the 148M allele influenced liver fat content independently of body mass index (BMI), dyslipidemia and insulin resistance, it has been demonstrated, in a group of obese adults, that morbid obesity exposes the association between the 148M allele and ALT. The degree of obesity seems, therefore, to act as a stressor on a specific genetic background (the 148M polymorphism) influencing the susceptibility to deleterious consequences (increased liver enzymes) [18]. Although the use of PNPLA3 genotyping in clinical practice, currently, appears controversial, the possibility exists that the use of PNPLA3 analysis in combination with non-genetic factors would really help in the prediction of individuals at risk for liver damage [17].

Aims of this study were i) to analyse in a large group of obese Italian children the potential relative role of the interaction of not-genetic factors such as BMI, waist circumference and insulin resistance in exposing the association between the I148M polymorphism and ALT levels and ii) to stratify the individual risk of these children to have liver injury on the basis of this gene-environment interaction.

## Materials and Methods

### Cohort description and clinical evaluation

Of 2720 Caucasian obese children and adolescents, consecutively referred to our ward (childhood obesity service) since the 1999, 1058 patients, randomly chosen, have been enrolled. No differences in mean age, sex distribution, pubertal stage and obesity degree were observed between the study sample and the sample of subjects not included in the study.

Patients had between 2 and 16 years of age and showed a BMI exceeding the 95^th^ percentile for their age and sex. Subjects using medications that alter blood pressure, glucose or lipid metabolism were excluded (10 patients). Children had undergone hepatitis B vaccination, which is compulsory in Italy. No prior history of hepatitis was reported in any children. The ethical committee of the Second University of Study of Naples approved the study. Written consent was obtained for each participating child from the parent/legal guardian of the child.

Of the 1048 subjects definitively enrolled in the study 525 were girls. This sample was representative of the 2720 children referred to our ward from 1999 to 2009. Weight and height were measured and BMI was calculated. Standard deviations scores (SDS) for BMI were calculated by using the LMS method [19]. The population mean age was 10.6±3 years; the mean BMI-SDS was 3±0.7. Pubertal stage was assessed using Tanner criteria [20].

Waist circumference was measured by trained technicians to the nearest centimeter with a flexible steel tape measure while the subjects were standing, after gently exhaling, as the minimal circumference measurable on the horizontal plane between the lowest portion of the rib cage and iliac crest [21]. The average value of 2 waist measurements was obtained and, as indirect measure of the amount of abdominal fat, the ratio between waist and height (W/Hr) was calculated.

### Metabolic evaluation

After informed consent, a blood sample was drawn from each patient after an overnight fast. The serum was frozen at −20°C until analysed. Triglycerides levels were determined by an enzymatic colorimetric test with lipid clearing factor. Serum ALT and AST were assayed using a Hitachi Analyser (Boerhinger-Mannheim Diagnostics, Indianapolis, IN). ALT greater than 40 U/L was classified as elevated [22]. In the children with elevated liver enzymes the presence of hepatitis C was excluded.

A subgroup of 497 children underwent an oral glucose tolerance test (OGTT). Insulin and glucose levels were measured during the OGTT at baseline and later every 30 minutes for 120 minutes.

Insulin resistance was assessed using the homeostasis model assessment (HOMA-IR). For a better definition of peripheral insulin sensitivity we calculated also the whole body insulin sensitivity index (WBISI). The composite WBISI is based on values of insulin and glucose obtained from the OGTT, as originally described [23] and represents good estimates for clamp-derived insulin sensitivity [24]. Immunoreactive insulin was assayed by IMX (Abbott Diagnostics, Santa Clara, CA). The mean intra- and inter-assay coefficients of variations were 4.7% and 7.2%, respectively.

### Genotyping

Patients were genotyped for PNPLA3 rs738409 C to G variant, underlying the I148M substitution, using in all cases both direct sequencing and restriction enzyme analysis. Samples giving discordant results were re-analyzed.

The following primers were used, F: 5′-GCCCTGCTCACTTGGAGAAA-3′ and R: 5′-TGAAAGGCAGTGAGGCATGG-3′. For restriction enzyme analysis *Fok*I enzyme was used to identify the variant, since the G allele eliminates a *Fok*I restriction site.

### Statistical analysis

A Chi Square test was used to verify whether the genotypes were in Hardy-Weinberg equilibrium and to compare categorical variables. Differences among genotypes for continuous variables were evaluated by a general linear model (GLM). When it was appropriate age, gender and BMI-SDS were used as covariates. Levene's test of equality of variance was used to test the differences of the variance of the quantitative trait ALT according to the PNPLA3 genotype. This test is based on the fact that, under plausible scenarios of gene-gene or gene-environment interaction, the variance of a quantitative trait (e. g.; ALT levels) is expected to differ among the three possible genotypes of a biallelic SNP (e. g.; PNPLA3) [25]. A comparison of regression lines was performed to examine the influence of the genotype on the relationship between ALT and W/Hr, BMI-SDS or HOMA-IR.

Not-normally distributed variables were log transformed before the analysis, but raw means are shown.

To evaluate which value of W/Hr had the best sensitivity/specificity ratio to predict pathologic ALT levels, we calculated a Receiver Operating Characteristic (ROC) curve. The area under the curve (AUC) measures the degree of separation between an affected and a non-affected subject by a specific test. An AUC of 1 indicates perfect separation between affected and non-affected subjects, whereas an AUC of 0.5 indicates no discrimination between the test values. The optimal cut-off point was obtained using the Youden index (maximum [sensitivity +specificity –1]).

A logistic regression was performed to calculate the odds of showing pathologic levels of ALT according to both the phenotype (i. e.; W/Hr) and the genotype (i. e.; PNPLA3 I148M polymorphism). Age, gender and BMI SDS were used as covariates.

The Stat-Graph 3.0 software for Windows was used for all the statistical analyses. ROC curve analysis was made using Statistical Program for Social Sciences Version 13.0 (SPSS Inc, Chicago, Ill). All data are expressed as means ± SD. P-values less than 0.05 were considered statistically significant and where appropriate were adjusted for multiple comparisons.

## Results

The frequency of the different *PNPLA3* rs738409 genotypes distribution was in Hardy-Weinberg equilibrium (p: 0.11).

Five hundred and thirty-one patients were homozygous for the wild type allele (CC), 415 were CG and 102 were GG (i.e.; both alleles producing the 148M protein). The frequency of the *PNPLA3* minor allele was 0.29, which is in line with the frequencies reported in obese subjects belonging to the same geographic area [18], [26]. The clinical characteristics of the study subjects are shown in Table 1.

**Table 1:** Clinical and laboratory characteristics of the 1048 children involved in the study.

**N**	1048
**Male/Female**	523/525
**Tanner 1 (%)**	50.3
**Tanner 2 (%)**	20
**Tanner 3 (%)**	15
**Tanner 4 (%)**	12.7
**Tanner 5(%)**	2
**Age (years)**	10.6±3
**BMI**	31±4.8
**BMI-SDS**	3±0.7
**W/Hr**	0.62±0.06
**HOMA**	5.5±4.3
WBISI*	2.5±1.6
**Total Cholesterol (mg/dl)**	160±31
**Triglycerides (mg/dl)**	99±49
**HDL-C (mg/dl)**	46±12
**ALT (U/L)**	29±22
**AST (U/L)**	24±9
**Gamma-GT (U/L)**	19±9

Values are expressed as means ± standard deviations. Ranges are in brackets. Abbreviations: BMI-SDS: Body Mass Index Standard Deviation Scores; W/Hr: Waist circumference to height ratio; HOMA-IR: homeostatic model of assessment of insulin resistance index; WBISI: whole body insulin sensitivity index; HDL-C: high density lipoprotein-cholesterol; ALT: alanine transaminase; AST: aspartate transaminase; Gamma-GT: Gamma-Glutamyl transferase. * WBISI was available in 497 patients.

We observed a striking increase in circulating ALT and AST levels in the children carrying the 148M allele (p = 0.000006 and p = 0.0002 respectively), adjusting for age, gender, pubertal stage, and BMI-SDS (Table 2). Thirty three percent of 148M homozygous had ALT levels above 40 U/L compared with 17% of heterozygous and 13% of homozygous for the major allele (p = 0.00001). Consistently, children homozygous for the 148M allele had an Odds Ratio (OR) to show pathologic ALT of 2.97 (95% C.I. 1.80–4.18, p = 0.00001) compared to the patients homozygous for the 148I allele. No differences for BMI-SDS, total cholesterol, HDL-C, triglycerides, HOMA and WBISI were found across the genotypes (Table 2).

**Table 2:** Clinical and laboratory characteristics of obese patients subdivided by PNPLA3 I148M genotype.

	II	IM	MM	P values
**Number (%)**	531 (51%)	415 (39%)	102 (10%)	
**Age (years)**	10.6±3	10.4±3	10.4±2.8	0.6
**BMI -SDS**	3±0.7	3±0.8	2.9±0.6	0.2
**W/Hr**	0.62±0.05	0.62±0.06	0.61±0.04	0.2
**HOMA-IR**	5.7±4.6	5.4±3.7	5.2±3.6	0.7
**WBISI** *	2.4±1.5	2.6±1.6	2.7±1.7	0.6
**Total Cholesterol (mg/dl)**	159±30	161±30	160±32	0.7
**Triglycerides (mg/dl)**	97± 44	100± 55	101±45	0.4
**HDL-C (mg/dl)**	46±12	46±12	46±11	0.8
**ALT (U/L)**	25±16	30±22	38±30	0.000006
**AST (U/L)**	23±8	25±10	28±10	0.0002
**Gamma-GT (U/L)**	18±8	19±9	19±10	0.4

Values are expressed as means ± standard deviations. GLM analysis including gender, age and pubertal stage as covariates has been used to compare continuous variables. Abbreviations: BMI-SDS: Body Mass Index Standard Deviation Scores; W/Hr: Waist circumference to height ratio; HOMA: homeostatic model of assessment of insulin resistance index; WBISI: whole body insulin sensitivity index; HDL-C: high density lipoprotein-cholesterol; ALT: alanine transaminase; AST: aspartate transaminase; Gamma-GT: Gamma-Glutamyl transferase.

*WBISI was available in 497 patients.

**Figure 1:**
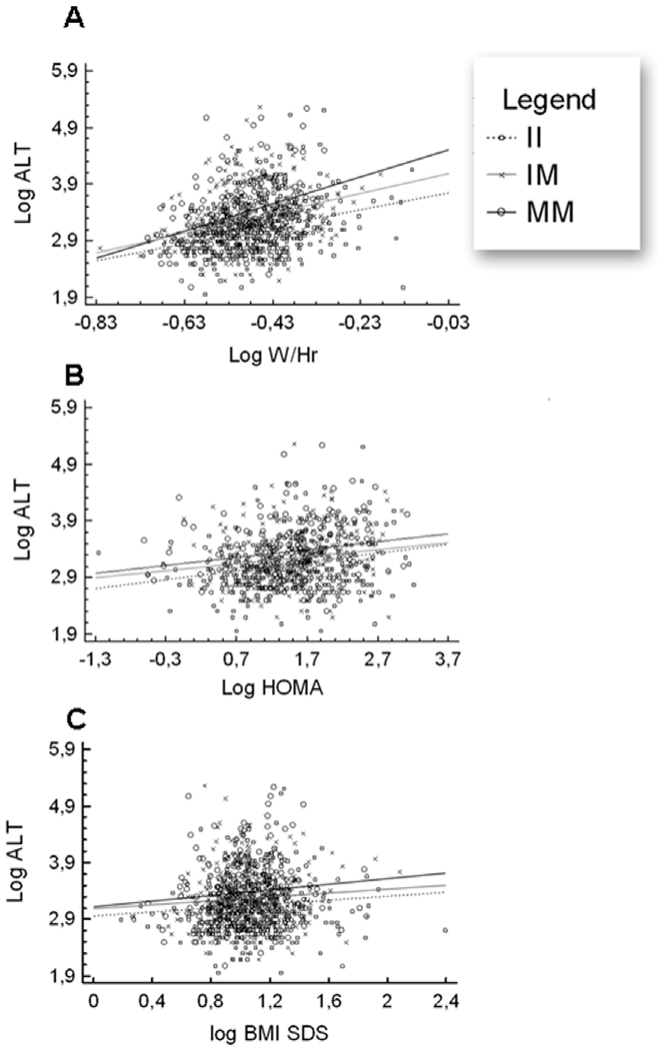
Association between ALT levels and W/Hr, HOMA and BMI-SDS according to the PNPLA3 genotype. **A**: Regression analysis describing the relationship between ALT levels and W/Hr in patients homozygous for *PNPLA3* M variant, heterozygous, and homozygous for PNPLA3 I variant. The regression between ALT levels and W/Hr in the group of patients homozygous for *PNPLA3* M/M is described by the equation y = 4.6+2.4*× (r = 0.36; p = 0.00001). The equation for *PNPLA3* I/M was y = 3.7+1.4*× (r = 0.22; p = 0.00001). The equation for PNPLA3 I/I was y = 3.4+1.1*× (r = 0.17; p = 0.0005). The comparison between the three regression lines is significant (p = 0.0045). **B**: Regression analysis describing the relationship between ALT levels and HOMA-IR in patients homozygous for *PNPLA3* M variant, heterozygous, and homozygous for PNPLA3 I variant. The regression between ALT levels and HOMA-IR in the group of patients homozygous for *PNPLA3* M/M is described by the equation y = 3.2+0.14*× (r = 0.18; p = 0.02). The equation for *PNPLA3* I/M was y = 3.0+1.12*× (r = 0.16; p = 0.001). The equation for PNPLA3 I/I was y = 2.9+0.16*× (r = 0.23; p = 0.00005). The three equations are not significantly different as to slopes (p = 0.7). **C**: Regression analysis describing the relationship between ALT levels and BMI z-score in patients homozygous for *PNPLA3* M variant, heterozygous, and homozygous for PNPLA3 I variant. The regression between ALT levels and BMI z-score in the group of patients homozygous for *PNPLA3* M/M is described by the equation y = 3.1+0.24*× (r = 0.01; p = 0.13). The equation for *PNPLA3* I/M was y = 3.1+0.17*× (r = 0.08; p = 0.14). The equation for PNPLA3 I/I was y = 2.9+0.17*× (r = 0.09; p = 0.04). The three equations are not significantly different as to slopes (p = 0.5).

Based on the theoretical observation that the within genotype variance of a quantitative trait will vary when a genetic or an environmental interaction is present, we explored the presence of an interacting covariate on the genetic effect of PNPLA3 on serum ALT levels. Levene's test of equality of variance was statistically significant (P<0.00001) strongly suggesting the presence of an interacting covariate. Among the biologically plausible environmental interacting factors, we focused on BMI-SDS, HOMA-IR and W/Hr. As expected, all these factors directly correlated with ALT levels in our population (BMI-SDS: r = 0.07, p = 0.01; HOMA-IR: r = 0.019, p = 0.00001; W/Hr: r = 0.25, p = 0.00001). A multivariate analysis including BMI-SDS, HOMA-IR and W/Hr in the model showed that the strongest predictor for ALT levels was W/Hr (p = 0.000001). Relationships between BMI-SDS, HOMA-IR and W/Hr with ALT levels were further analysed in function of the different PNPLA3 genotypes, comparing the slope of the relative regression lines (Figure 1).

Children homozygous for the *PNPLA3* minor allele (148M) showed a stronger correlation between ALT and W/Hr than those carrying theI/M or the I/I genotypes, as resulting by the comparison of regression lines (p: 0.0045) (Figure 1. A), supporting the idea that the effect of rs738409 on ALT levels has a relevant magnitude overall in conditions of excess abdominal fat accumulation. Difference among the three regression line intercepts was statistically significant (p<0.01).

Comparison of the regression line slopes was performed for BMI-SDS and HOMA-IR and was not significant (Figure 1. B and 1. C).

To identify children at greater risk to have pathologic ALT levels on the basis of the size of their abdominal fat, we performed a ROC curve analysis to detect the W/Hr with the best sensitivity and specificity, which resulted 0.62 (AUC = 0.624; 95% CI = 0.58–0.67; Sensitivity = 0.665, Specificity = 0.541, p = 0.0001**)**. Successively, in order to obtain a complete risk stratification of the possibilities to have liver injury, we subdivided the population of obese children in six categories according to W/Hr (below or above 0.62) and to PNPLA3 genotypes (I/I, I/M or M/M) (Table 3).

**Table 3:** Risk of pathologic ALT levels in 1048 obese children stratified in six categories according to fat abdominal size (W/Hr) and PNPLA3 genotype.

Categories	W/Hr	PNPLA3 Genotype	Patients number	ALT (UI/L)	Patients (%) with ALT>40 UI/L	OR (C.I.) p value
I	>0.62	MM	47	48±34	51%	4.9 (3.2–7.8) 0.00001
II	>0.62	IM	203	32.9±23	20.3%	1.5 (0.9–3.8) 0.06
III	>0.62	II	271	29±20	17.6%	1.01 (0.7–1.5) 0.9
IV	≤0.62	MM	55	30±21	18.2%	1.2 (0.7–2.4) 0.1
V	≤0.62	IM	212	28.0±18	15.9%	0.8 (0.4–1.5) 0.6
VI	≤0.62	II	260	22.7±11	6.7%	0.28 (0.1–0.5) 0.00001

ALT (alanine transaminase) values are expressed as means ± standard deviations. Logistic regression analysis has been used to calculate the Odds Ratios (OR) to have ALT>40 UI/L for each category of patients (from I to VI), compared to the entire cohort of children.

Logistic regression analysis showed that subjects homozygous for the PNPLA3 minor allele and with W/Hr above 0.62 (category I) had the highest OR (4.9, 95% C. I. 3.2–7.8, p = 0.00001) to develop pathologic ALT and that OR progressively reduced in the other groups of patients according to the strength of the interaction between W/Hr and PNPLA3 genotype (Table 3).

In fact, children homozygous for the PNPLA3 wild allele (148I) and with W/Hr equal or below 0.62 (category VI) showed the lowest possibilities to have pathologic ALT (OR 0.28, 95% C. I. 0.1–0.5, p = 0.00001), (Table 3). The post-hoc statistical power for multiple regression of the present study is 0.99. Liver enzymes of one hundred and fifty patients who did not enter in a weight loss program and were, therefore, still obese, were re-evaluated after 5 years. Using the categories assigned at the time of the first evaluation we observed the persistence of the difference concerning ALT levels. Particularly, patients who had belonged to category I showed the highest mean ALT levels (53±28 UI/L), and ALT levels progressively decreased from category I to category VI (p = 0.01).

## Discussion

Two recent studies have shown an association between the rs738409 polymorphism in PNPLA3 and increased liver enzymes in Caucasian obese subjects, both adults and children [18], [26]. These data were in agreement with previous results obtained in Hispanics [9], an ethnic group with high propensity to develop liver steatosis and inflammation. In both cases the polymorphism was not associated with crude estimates of insulin resistance. In other cohorts of African American, European American and European subjects, not selected on the basis of their BMI, the association between PNPLA3 polymorphism and liver enzymes has not been found [9], [27].

In line with the data on obese subjects, we report in a group of 1048 Italian obese children and adolescents that the PNPLA3 rs738409 polymorphism was associated with ALT levels in a dose-dependent manner.

We expanded the knowledge about the lack of association between the polymorphism and insulin resistance investigating whether the PNPLA3 148M allele was associated with insulin sensitivity estimated from OGTT. The advantage of this method for evaluating insulin sensitivity compared to the use of fasting values, as previously done [18], [26], is that it provides a more dynamic and precise measurement of this phenotype [27]. We found no relationships of the PNPLA3 polymorphism with insulin sensitivity, in agreement with similar results obtained in obese adults [18] and with a recent work showing, in obese children subjected to an insulin clamp, that the level of hepatic and peripheral insulin resistance are not associated to PNPLA3 rs738409 polymorphism [28]. Furthermore, we found no association with BMI and serum triglycerides. Although a selection bias, considering that about one half of the investigated children is in different pubertal state, may be suspected, it should be considered that all analyses have been adjusted for pubertal development.

Pediatric population represents an ideal group of subjects to study the interaction between genetic factors and phenotypic characteristics in producing liver injury because of the low number of confounding factors in pediatric patients (e. g.; the duration of the disease, lifestyle habits, comorbidities, and drugs).

Based on this concept, we have investigated for the first time if obesity degree, insulin resistance or abdominal fat might modulate the strength of the effect of PNPLA3 I148M polymorphism on liver enzymes and we have found a strong interaction between W/Hr and PNPLA3 genotype. This suggests that waist circumference may play an important role in the measure of the effect of the I148M polymorphism on ALT levels.

The clinical use of waist circumference in children is limited by the lack of an internationally accepted classification which gives age-specific waist circumference cutoffs and by the lack, in most countries, of population-based reference values [29]. To overcome these limitations the use of the waist-to-height ratio has recently been proposed [30]–[33]. Waist circumference is highly correlated with visceral adipose tissue and metabolic and cardiovascular risk factors in children, adolescents and adults [34], [35]. This support the idea that the effect of the I148M variant on liver enzymes has a relevant magnitude in condition of excess of visceral fat which, therefore, acts as a stressor on a specific genetic background.

That waist circumference, rather than generalised obesity, contributes to liver damage in children with NAFLD has been demonstrated analysing liver biopsies of about two hundred Caucasian children [7]. Furthermore, examining the association between visceral fat evaluated by magnetic resonance and liver inflammation in patients with NAFLD van der Poorten et al. have showed that the extent of liver injury augmented with increase in visceral fat and this correlation remained statistically significant even when controlled for insulin resistance and hepatic steatosis [36]. Therefore, visceral fat appears directly associated with liver inflammation, independently of insulin resistance and hepatic steatosis. Recently, an association between the PNPLA3 I148M polymorphism and histologic liver damage (NASH and liver fibrosis) has been reported [13]–[15]. In one of these reports [13] it has been shown that adiponutrin 148M variant may modulate the expression of molecules, such as peroxisome proliferator-activated receptor-alpha (PPAR-alpha) and fas ligand (FASL) [37] implicated in the pathogenesis of liver injury [38]. Putting these data together with our results we speculate that the exposing role played by abdominal fat on the PNPLA3 polymorphism may be sustained by the close direct association between visceral fat and liver inflammation. Nevertheless, the possibility that, according to the data of Abate et al. [39], [40], is the abdominal subcutaneous fat rather than the visceral fat to play a major role in this association, based on our results, cannot be dismissed.

Feldstein et al. have recently demonstrated that NAFLD in children is associated with a significantly shorter survival as compared to survival of the general population of same age and sex [4]. Furthermore, it has been shown that average annual overall health care costs are significantly higher for individuals with fatty liver disease and increased ALT levels compared to the general populations [41]. It would be, therefore, of great clinical value to identify those obese children at higher risk for NAFLD who would be expected to benefit the most from medical therapy.

The stratification of the entire population of obese children on the basis of their PNPLA3 genotype and of the size of abdominal fat (W/Hr) in six groups with increasing possibilities to show pathologic liver enzymes adds novel and original knowledge in the field and would represent an important step in the stratification of the individual risk to have liver injury.

Concluding, we have studied the PNPLA3 I148M polymorphism in the largest cohort of obese children till now investigated and we have for the first time i) showed that the magnitude of the association with liver enzymes is driven by the size of abdominal fat and ii) stratified the individual risk of these patients to develop liver damage on the basis of the interaction between the PNPLA3 genotype and the size of abdominal fat.
